# On the Faculties Possessed by Certain Elements of the Blood, of Regenerating the Vital Properties

**Published:** 1856-04

**Authors:** E. Brown-Sequard


### ON THE FACULTY POSSESSED BY CERTAIN ELEMENTS OF THE BLOOD, OF REGENERATING THE VITAL PROPERTIES.

BY E. BROWN-SEQUARD, M. D.

At the session of the *Academie des Sciences*, held in October last, Dr. Brown-Sequard detailed a great number of experiments, showing that the contractile and nervous tissues, after having entirely lost their vital properties in consequence of a complete interruption of the circulation of the blood, may recover these properties under the influence of red blood injected into the arteries of these tissues. He remarks:

In a single experiment, it is possible to observe at once almost all the facts above related, as regards the restoration of vital powers in muscles, and in motor and sensitive nerves.

A ligature is put around the aorta, behind the origin of the renal arteries, in a strong rabbit. Voluntary movements, sensibility, reflex actions, the power of motor nerves, and, at last, muscular contractility, successively disappear from the posterior limbs of this animal. I wait until cadaveric rigidity has come, and even until it exists in all the parts of these limbs, and then I take away the ligature. The anterior part of the body of the rabbit being still living, circulation recommences in the posterior part of the body, and, with the arrival of blood in that part, muscular contractility, the power of the motor nerves, reflex actions, sensibility, and, at last, voluntary movements successively re-appear in the posterior limbs. From this experiment, it results that, not only local life, but the properties and actions of full life, can be restored in limbs that have been in the state called rigor mortis, cadaveric, or post mortem rigidity.

Since the 9th of June, 1851, when I communicated to the *academie* the details of the first experiments of this kind that I have made, I have performed them many times, and they have been repeated with success by Professor Stannius, of Rostock.

Dr. Brown-Sequard has found that the muscular tissues, the motor and sensitive nerves and the spinal cord will recover their vital properties under the influence of red blood injected into their vessels. The contractile fibres around the hair bulbs, which produce the appearance called cutis anserina, may, under this influence, recover their contractility long after they have lost it. In an experiment on a man guillotined in 1851, the cutis anserina was produced on the fore arm more than twenty-four hours after decapitation, by injecting blood into the brachial artery.

It was found that blood, which had been deprived of its fibrine, would restore the vital properties of these tissues as quickly as that still containing it. This important fact might lead us to doubt the agency of fibrine in the process of nutrition. Experiment also proved that the serum of the blood did not possess this regenerating faculty. Dr. Brown-Sequard concludes that it is principally the oxygen of the blood which restores the vital properties of the tissues, although he does not deny the agency of the blood globules and albumen in aiding the result. He considers that it is a part of the functions of the blood globules to absorb oxygen and carry it to the tissues, and he has, by artificially oxygenating venous blood, produced the phenomena of re-exciting the vital properties of the muscular and nervous tissues.

The contractility of muscles can be reproduced three or four times in the same animal by these injections of oxygenized blood; and the laborious experimenter declares that he has maintained the contractility of a muscle separated from the body of a rabbit for fifteen hours, when fatigue compelled him to stop the experiment. The following are his conclusions:

1. The contractile tissues, i. e. the muscles of animal life, the muscular fibres of the uterus, of the bladder, of the heart, of the bowels, of the iris, of the skin, may, under the influence of red and defibrinated blood, recover their contractility, from half an hour to five hours after having lost it.

2. The motor and sensitive nerves, and the spinal cord, may also, under the same influence, recover their vital properties, from a quarter of an hour to about an hour after having lost them.

3. The vital properties appear to be independent one from another, and their existence seems to depend upon the organization of the tissues, and upon the maintenance of this organization by red blood.

